# Supplementary material for: Sustained-input switches for transcription factors and microRNAs are central building blocks of eukaryotic gene circuits
Source: Genome Biol. 2013 Aug 23;14(8):R85. doi: 10.1186/gb-2013-14-8-r85 (PMC4054853; doi:10.1186/gb-2013-14-8-r85)
Supplement: Additional file 5 — HTML Browsable Motif Output. Zipped folder containing all WaRSwap and FANMOD motif output, viewable in a web browser. [file gb-2013-14-8-r85-S5.ZIP › HTML_browsable_motif_output/WaRSwap_all_tair9/sigs_WSWRSLDraw-2000_all.pvals.heatmaps.html/motif_id_46_011101001_tftype_all_upstream_-1000_0.html]

```
BG_MODEL = Rnew2
MOTIF_ID = 46_011101001
TF_TYPE = all
UPSTREAM = -1000_0


PVals
FN_0.2	FN_0.4	FN_0.6	FN_0.8
dg_60.genes	0.003	0.004	0.05	0.492
dg_70.genes	0.003	0.004	0.052	0.496
dg_80.genes	0.003	0.005	0.049	0.479

ZScores
FN_0.2	FN_0.4	FN_0.6	FN_0.8
dg_60.genes	2.821	2.686	1.78	-0.346
dg_70.genes	2.796	2.595	1.706	-0.359
dg_80.genes	2.796	2.623	1.707	-0.331

StDevs
FN_0.2	FN_0.4	FN_0.6	FN_0.8
dg_60.genes	6064.584	1158.252	89.678	3.886
dg_70.genes	6156.51	1179.514	90.045	3.721
dg_80.genes	6105.345	1201.756	91.576	3.923
```
